# Determinants in the LIN-12/Notch Intracellular Domain That Govern Its Activity and Stability During *Caenorhabditis elegans* Vulval Development

**DOI:** 10.1534/g3.116.034363

**Published:** 2016-09-16

**Authors:** Yuting Deng, Iva Greenwald

**Affiliations:** * Department of Biological Sciences, Columbia University, New York 10027; † Department of Biochemistry and Molecular Biophysics, Columbia University, New York 10027

**Keywords:** Notch, *C. elegans*

## Abstract

Upon ligand binding, the LIN-12/Notch intracellular domain is released from its transmembrane tether to function in a nuclear complex that activates transcription of target genes. During *Caenorhabditis elegans* vulval development, LIN-12/Notch is activated by ligand in two of six multipotential vulval precursor cells (VPCs), specifying the “secondary vulval fate” and descendants that contribute to the vulva. If LIN-12 is ectopically activated in other VPCs, they also adopt the secondary fate, dividing to produce extra vulval cells, resulting in a “Multivulva” phenotype. Here, we identify determinants in the LIN-12 intracellular domain [“LIN-12(intra)”] that govern its activity and stability during *C. elegans* vulval development; we assayed activity of mutant forms based on their ability to cause a Multivulva phenotype and stability using a GFP tag to visualize their accumulation. Our analysis has revealed that, while the ubiquitin ligase SEL-10/Fbw7 promotes LIN-12(intra) downregulation in VPCs, there is a distinct mechanism for downregulation of LIN-12(intra) in VPC descendants. Our analysis also revealed that LIN-12(intra) must be in the nuclear complex to be regulated appropriately in VPCs and their descendants, and that the structure or conformation of the carboxy-terminal region influences stability as well. Although activity and stability are generally well-correlated, exceptions where they are uncoupled suggest that there may be roles for the carboxy-terminal region and *sel-10* that are independent of their roles in regulating LIN-12(intra) stability.

LIN-12/Notch is a transmembrane protein receptor that is cleaved upon ligand binding to release the intracellular domain from a membrane tether. The liberated intracellular domain forms a nuclear complex with a sequence-specific DNA binding protein called CSL (CBF1, Suppressor of Hairless, LAG-1) and an additional protein, Mastermind (SEL-8 in *Caenorhabditis elegans*). This nuclear complex activates the transcription of target genes.

The activity and stability of the LIN-12/Notch intracellular domain is regulated during normal development and tissue homeostasis, and abrogation of such regulation can cause disease (Aster *et al.* 2008; Belver and Ferrando 2016). This regulation is achieved by the interaction of modulatory factors with determinants within the LIN-12/Notch intracellular domain. The best example is afforded by SEL-10/Fbw7 regulation of LIN-12/Notch in *C. elegans* and mammals. *sel-10* was found as a negative modulator of LIN-12/Notch through genetic analysis in *C. elegans* (Sundaram and Greenwald 1993; Hubbard *et al.* 1997); its ortholog, Fbw7, was subsequently found to be a tumor suppressor that contributes to T-cell acute lymphoblastic leukemia (T-ALL) via negative modulation of Notch1 (Oberg *et al.* 2001; Wu *et al.* 2001; Gupta-Rossi *et al.* 2001). SEL-10/Fbw7 is the substrate recognition component of a multiprotein E3 ubiquitin ligase that acts via a conserved sequence in the intracellular domain called a Cdc4 phosphodegron (CPD); phosphorylation of the CPD of LIN-12/Notch recruits SEL-10/Fbw7, leading to ubiquitination and degradation of the intracellular domain. Mutation or deletion of the CPD increases stability and activity of the intracellular domain, which causes developmental abnormalities in *C. elegans* (Mango *et al.* 1991; Hubbard *et al.* 1997; Li and Greenwald 2010; de la Cova and Greenwald 2012), and in humans causes T-ALL (O’Neil *et al.* 2007; Thompson *et al.* 2007).

Here, we analyzed determinants affecting the stability of the LIN-12 intracellular domain [LIN-12(intra)] in the vulval precursor cells (VPCs), a *C. elegans* developmental paradigm that has provided many insights into the role of Notch in mediating binary cell fate decisions during development and the mechanism of signal transduction by Notch (reviewed in Greenwald 2012). Our analysis has revealed that, while *sel-10* promotes LIN-12(intra) downregulation in VPCs, there is a distinct mechanism for downregulation of LIN-12(intra) later, after VPC fate specification and cell division. Both mechanisms require association with LAG-1/CSL in the nuclear transcription complex and a previously undefined structural motif in the carboxy-terminal region, which we term the “Y region.” Our analysis further suggests that there may be roles for the carboxy-terminal region and SEL-10/Fbw7 that are independent of their roles in regulating LIN-12(intra) stability.

## Materials and Methods

### Genetic analysis

The *C. elegans* Bristol strain N2 was used as the wild type in this study. The LGIII mutation *pha-1(e2123ts)* and LGV mutation *sel-10(ar41)* were used. A complete list of strains with full genotypes is provided in Supplemental Material, Table S3. All strains were grown at 25°.

To assess the Multivulva phenotype, we picked individual L4 adults containing the relevant extrachromosomal array, and scored for the Multivulva phenotype ∼24 hr later by inspection. Worms with ≥2 ectopic pseudovulvae were counted as exhibiting the Multivulva phenotype. To assess LIN-12 protein accumulation, worms carrying extrachromosomal arrays were mounted on 2% agarose pads on a slide, immobilized with 10 mM levamisole in M9, and analyzed using the 63 × objective on a fluorescent microscope at 600 msec exposure time in the GFP channel.

### Plasmid construction

Each LIN-12 construct plasmid was generated by inserting a PCR product of the LIN-12 segment into a plasmid containing regulatory sequences from the *lin-31* gene and the *unc-54* 3′ UTR (Tan *et al.* 1998). The plasmid p874 [*lin-31*p::*lin-12(intra)*] was digested with *Bgl*II and *Not*I, and the backbone containing *lin-31p* was isolated and used for all plasmid generation. Each LIN-12 segment was individually constructed via PCR and using the template p385 [*lin-12(cDNA)*::*GFP*]. Fusion PCR methods were used for some constructs. PCR fragments were digested with *Bgl*II and *Not*I, then inserted into the backbone containing *lin-31p* via FastLink ligation methods. The plasmids constructed and primers used are listed in Table S1 and Table S2.

### Construction of transgenic worms

All transgenes are complex arrays (Kelly *et al.* 1997) that were generated by selection for rescue in the pha-1(e2123) background (Granato *et al.* 1994). Each LIN-12 construct plasmid was linearized and injected at 1 ng/μl, along with pBX (*pha-1(+)*) and p716 (*myo-3p*::*mCherry)* at 1 ng/μl and *Pvu*II-digested N2 gDNA at 50 ng/μl, into 20 *pha-1(e2123ts)*. Injected P0s were kept at 15° for 3 d, then shifted to 25° for 4 d. Independent transgenic lines were isolated from F2s, generating a maximum of one line per injected P0. The transgenes generated are included in the genotypes of the strains listed in Table S3.

### Data availability

All strains and constructs are available upon request. Please request strains using the “GS” name or plasmids using the “P” designation as in Table S1 and Table S3. The authors state that all data necessary for confirming the conclusions presented in the article are represented fully within the article.

## Results

### Distinct mechanisms for temporal regulation of the stability of the LIN-12 intracellular domain in VPCs

**Figure 1:**
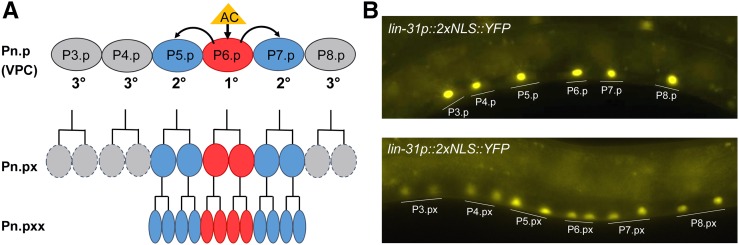
Wild-type vulval development and the heterologous expression system used. (A) Schematic representation of the six VPCs. The six VPCs are numbered P3.p–P8.p; their daughters (Pn.px) and granddaughters (Pn.pxx) are also indicated. Normally, P6.p adopts the primary fate associated with EGFR activation (red) and P5.p and P7.p adopt the secondary fate associated with LIN-12 activation (blue). The Pn.px cells produced by primary and secondary VPCs undergo additional rounds of division. The Pn.px daughters of tertiary VPCs fuse with the major hypodermal syncytium and are not present later. Most Pn.pxx cells undergo one further round of division (not shown). (B) Expression in VPCs. Heterologous regulatory sequences from the *lin-31* gene (Tan *et al.* 1998) can be used to drive expression of inserted cDNAs in VPCs (top) and their daughters (bottom). The expression system and strain shown in this photomicrograph are described further in de la Cova and Greenwald (2012). cDNA, complementary DNA; EGFR, epidermal growth factor receptor; VPC, vulval precursor cell.

We analyzed determinants affecting the stability of the LIN-12 intracellular domain [LIN-12(intra)] in the VPCs. The VPCs are the six polarized epithelial cells, numbered P3.p–P8.p, which have the potential to adopt one of three fates depending on the signaling inputs they receive in the L3 stage (Figure 1A; Sundaram 2005). During normal development, P6.p adopts the primary fate associated with activation of an EGF receptor-Ras-ERK cascade; P5.p and P7.p adopt the secondary fate associated with activation of LIN-12/Notch; and P3.p, P4.p, and P8.p adopt the default, tertiary fate as in the absence of signaling (Sternberg 2005). After specification, all six VPCs divide to generate daughters (“Pn.px”); the daughters of the primary and secondary VPCs divide further to produce vulval cells, whereas the daughters of the tertiary VPCs fuse with the major hypodermal syncytium (Figure 1A). Mutations in endogenous *lin-12* that cause strong, constitutive activation of LIN-12 cause all six VPCs to adopt the secondary fate, resulting in a “Multivulva” phenotype (Greenwald *et al.* 1983).

In this study, we used regulatory sequences derived from the *lin-31* gene and a heterologous 3′ UTR (based on Tan *et al.* 1998) to generate transgenes that express GFP-tagged forms of LIN-12(intra) in VPCs and their descendants (Figure 1B). Under these transgene conditions, wild-type LIN-12(intra)::GFP does not cause strong constitutive activation because it is not stable. Therefore, we can assess if mutant forms (i) have strong constitutive activity resulting in the characteristic “Multivulva” phenotype associated with ectopic secondary fate, and (ii) are stable based on GFP accumulation in VPCs and their descendants.

**Figure 2:**
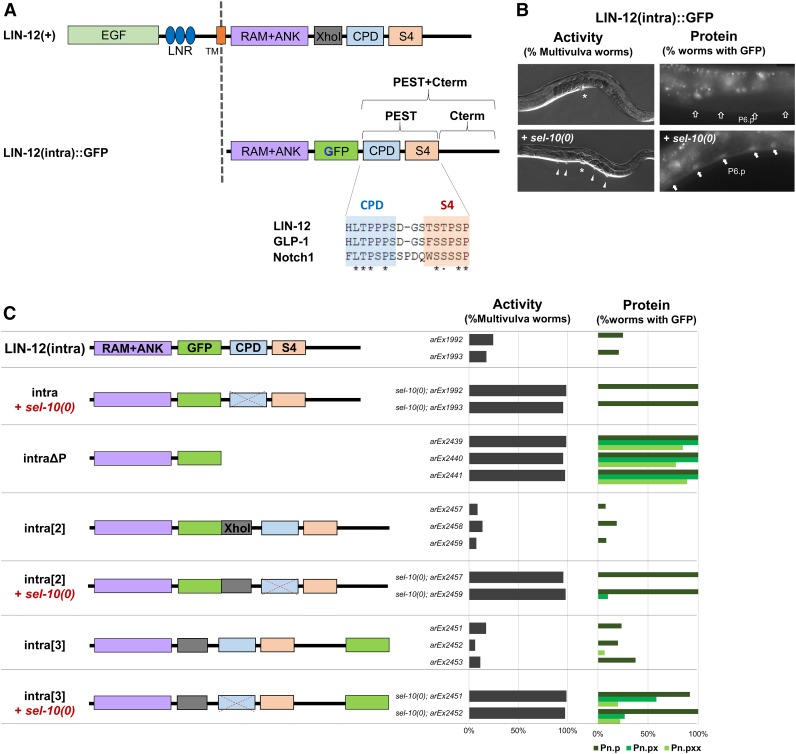
Distinct mechanisms for temporal regulation of the stability of the LIN-12 intracellular domain in VPCs. Here and in subsequent figures, two to three extrachromosomal lines were scored for each condition. For assaying activity, we scored *n* = ∼100 worms for each line for the Multivulva phenotype. For assaying protein stability, we looked at *n* = 10–20 worms at each stage [Pn.p (VPCs), Pn.px (their daughters), and Pn.pxx (their granddaughters)] for each line, with a 600 msec exposure time. (A) Schematic representation of LIN-12 forms. Full-length LIN-12 and LIN-12(intra)::GFP, the engineered, tagged canonical form used for most of the analysis in this paper, are shown in schematic form (not to scale). The “*Xho*I” segment of full-length LIN-12 has been replaced by GFP in the canonical LIN-12(intra)::GFP form. In (C), we show that this deletion has no consequence for the phenomena we describe here. Also shown are the amino acid sequences of the CPD and S4 domains of the PEST sequences from *C. elegans* LIN-12 and GLP-1, which can functionally substitute for LIN-12 in VPCs (Fitzgerald *et al.* 1993), and from human Notch1. (B) Photomicrographs of phenotypes scored to infer activity and stability. In an otherwise wild-type background, LIN-12(intra)::GFP does not cause an appreciable Multivulva phenotype or accumulate visible GFP in VPCs; however, removal of the negative regulator *sel-10*/Fbw7 causes a highly penetrant Multivulva phenotype and visible GFP accumulation. In every worm with visible GFP, fluorescence is present in most or all VPCs. We note that the expression system shown in Figure 1B allows for constitutive *lin-12* activity in the VPCs but, in contrast to constitutive mutations in the endogenous *lin-12* gene, the somatic gonad is not affected. Thus, EGFR is activated in P6.p; this signaling promotes the primary fate and blocks the effect of constitutive LIN-12, so a normal vulva is formed. The Multivulva phenotype reflects transformation of VPCs that would normally have adopted the tertiary fate so that they instead adopt the secondary fate. However, GFP fluorescence is seen in all six VPCs, including P6.p, consistent with previous observations that the EGFR-mediated block to nuclear LIN-12 is subsequent to nuclear import (Li and Greenwald 2010). (C) Testing the effect of the position of the GFP tag in LIN-12(intra)::GFP forms. In the canonical LIN-12(intra)::GFP, the GFP sequences replace a short segment of LIN-12 protein encoded by DNA flanked by *Xho*I sites, represented by the gray box. As shown in (A), GFP replaces the “*Xho*I” segment in the canonical form, labeled “intra” here, and this sequence is also lacking in the canonical LIN-12ΔP form that has high constitutive activity. Restoration of this *Xho*I segment, in form LIN-12(intra)::GFP[2], does not change the pattern of protein accumulation, indicating that the *Xho*I segment does not contain information that stabilizes LIN-12(intra) in VPC descendants. Placement of GFP at the extreme carboxy-terminus of full-length LIN-12(intra), in form LIN-12(intra)::GFP[3], may have a modest effect on downregulation. The dotted “X” over the CPD in a *sel-10(0)* background symbolizes the abrogation of negative regulation of stability through this domain. EGF, epidermal growth factor; CPD, Cdc4 phosphodegron; GFP, green fluorescent protein.

The domain structure of the LIN-12 intracellular domain is shown in Figure 2A. As described below, the amino terminal RAM+ANK domain, a hallmark of all Notch proteins, is sufficient to form the ternary complex with LAG-1 and SEL-8 (Wilson and Kovall 2006) and promote activation of LIN-12/Notch targets [(Li and Greenwald 2010); see also “LIN-12(intraΔP)” below]. Following the RAM+ANK domain is a PEST sequence; the term “PEST” has been applied in different ways to the carboxy-terminal region of Notch, but here we use it to indicate the sequence predicted by the program PESTFIND (Rice *et al.* 2000). In LIN-12, the PEST sequence consists of two motifs: the aforementioned CPD and the “S4” domain, both also present in mammalian Notch proteins. The CPD is the well-characterized phosphodegron targeted by SEL-10/Fbw7 (Welcker and Clurman 2008); mutational analysis of human Notch1 has shown that the S4 domain may work in conjunction with the CPD in regulating the stability of the intracellular domain (Chiang *et al.* 2006). We refer to the carboxy-terminal region following the PEST sequence in LIN-12 as “Cterm;” it is not overtly conserved in amino acid sequence between LIN-12 and mammalian Notch proteins but, as we show herein, we have identified a sequence within the Cterm that influences the stability of LIN-12(intra).

When we express intact LIN-12(intra) tagged with GFP in VPCs, we do not see ectopic secondary fate or visible GFP fluorescence; in contrast, *cis*-deletion of the PEST+Cterm region in the truncated form we have previously called LIN-12(intraΔP) results in strong constitutive activity, manifested as a Multivulva phenotype (Li and Greenwald 2010) (see Figure 2, B and C). Thus, there are negative regulatory elements in the PEST+Cterm region. One of these elements is the CPD; thus, removal of *sel-10*/Fbw7 also results in a Multivulva phenotype. However, deletion of PEST+Cterm is not equivalent to loss of *sel-10* in terms of the temporal profile of protein accumulation. In a *sel-10* null [*sel-10(0)*] background, LIN-12(intra)::GFP is stabilized only in VPCs but not their descendants; in contrast, when the PEST+Cterm sequence is deleted *in cis*, the resultant protein is stable in both VPCs and their descendants (Figure 2, B and C). These observations suggest that there are distinct mechanisms that govern the temporal stability of LIN-12(intra), and that a *sel-10*-independent pathway regulates LIN-12(intra) stability in the VPC descendants.

We were concerned that this temporal difference might be an artifact of the canonical LIN-12(intra)::GFP we first tested. In the canonical form, GFP replaces a sequence between two *Xho*I restriction sites (Figure 2A), as in the original GFP-tagged full-length form of LIN-12 that had been well-characterized for its activity and trafficking (Levitan and Greenwald 1995; Shaye and Greenwald 2002). Thus, we restored the sequence encoded by the *Xho* fragment to make LIN-12(intra)::GFP[2], such that GFP is now inserted into an otherwise intact LIN-12(intra) (Figure 2B). Three independent transgenic lines expressing LIN-12(intra)::GFP[2] had the same properties as transgenes that express canonical LIN-12(intra)::GFP: we did not observe a Multivulva phenotype, indicating that the form has low constitutive activity, or visible GFP expression in the VPCs, indicating that it is not stable. When we placed LIN-12(intra)::GFP[2] transgenes into a *sel-10(0)* background, the penetrance of the Multivulva phenotype was high and GFP was visible in VPCs, but not in their descendants. Thus, the temporal determinant that leads to degradation in VPC descendants was not present in the “missing” sequence, and resides in the PEST+Cterm region.

As a further test, we changed the position of the GFP tag to make LIN-12(intra)::GFP[3], which has an uninterrupted, intact intracellular domain with the GFP fused in-frame to the carboxy-terminus (Figure 2C). As with forms in which GFP is inserted within the intracellular domain, LIN-12(intra)::GFP[3] has low constitutive activity and low accumulation in VPCs in an otherwise wild-type background, and is enhanced in a *sel-10(0)* background to cause both a Multivulva phenotype and accumulation of GFP (Figure 2C). Although there may be modest stabilization in VPC descendants, further analysis described below revealed that the carboxy-terminus influences stability in VPC descendants, suggesting that, in LIN-12(intra)::GFP[3], the carboxy-terminal GFP tag may cause some stabilization.

In all subsequent experiments, the GFP tag is situated as in the canonical LIN-12(intra)::GFP protein.

### The CPD and S4 domains each negatively regulate stability of LIN-12(intra) in VPCs but do not affect stability of LIN-12(intra) in VPC descendants

We tested the role of the conserved amino acid residues in the PEST sequence for their effects on stability in VPCs and their descendants.

#### CPD:

**Figure 3:**
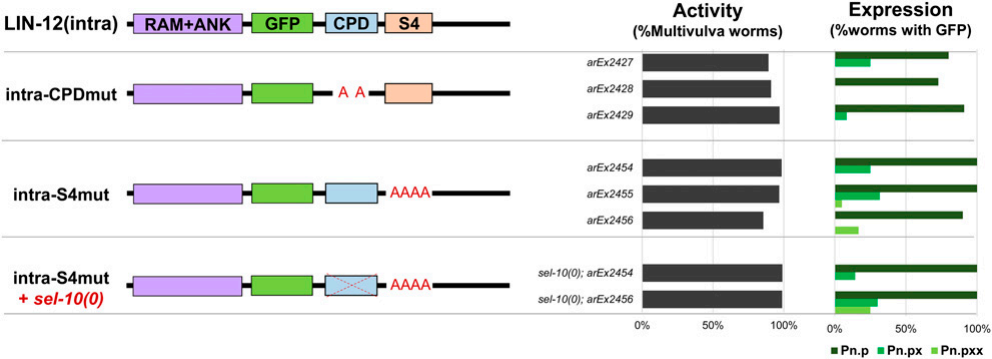
The CPD and S4 domains mediate stability of LIN-12(intra) in VPCs but do not mediate stability of LIN-12(intra) in VPC descendants [Pn.p (VPCs), Pn.px (their daughters), and Pn.pxx (their granddaughters)]. Mutation of either the CPD or S4 domain prevents degradation of LIN-12(intra), increasing LIN-12(intra) activity and protein stabilization in the VPCs. Neither the CPD nor S4 domain contain the regulatory information for protein downregulation in the VPC descendants, and loss of *sel-10* does not affect LIN-12(intra-S4mut) stabilization in the VPC descendants. CPD, Cdc4 phosphodegron; GFP, green fluorescent protein.

LIN-12(intra)::GFP is stable only in VPCs, but not their descendants, in a *sel-10(0)* background. Mutation of the critical CPD residues—the “central threonine” and “+ 4 serine” (Welcker and Clurman 2008)—to alanine in LIN-12(intra-CPDmut) mimics the behavior of a full length LIN-12(intra) in a *sel-10(0)* background, with strong a Multivulva phenotype (de la Cova and Greenwald 2012) and visible GFP in the VPCs but not in their descendants (Figure 2B and Figure 3). This observation supports the inference that *sel-10* does not mediate the downregulation of LIN-12(intra) in VPC descendants.

#### S4:

The S4 domain is a stretch of four serines immediately after the CPD of human Notch1; mutation of these serines to alanine stabilizes the Notch intracellular domain (Chiang *et al.* 2006). *C. elegans* LIN-12 and its paralog, GLP-1, also have a putative S4 region (Figure 2A), and mutation of the S4 serines and threonine of LIN-12(intra) to alanines [LIN-12(intra-S4mut)] causes a strong Multivulva phenotype and visible GFP in the VPCs, but not in their descendants (Figure 3). Thus, the S4 domain is not required for *lin-12* downregulation in the VPC descendants.

To test if *sel-10*-mediated downregulation, which occurs via the CPD, is redundant with the S4 domain for downregulation of LIN-12(intra) in VPC descendants, we crossed LIN-12(intra-S4mut) transgenes into a *sel-10(0)* background. We did not observe stabilization of the mutant protein in VPC descendants (Figure 3), suggesting that the CPD and the S4 domains are not functionally redundant determinants for stability in VPC descendants.

### The structure of the carboxy-terminal region influences stability of LIN-12(intra) in VPCs and their descendants

**Figure 4:**
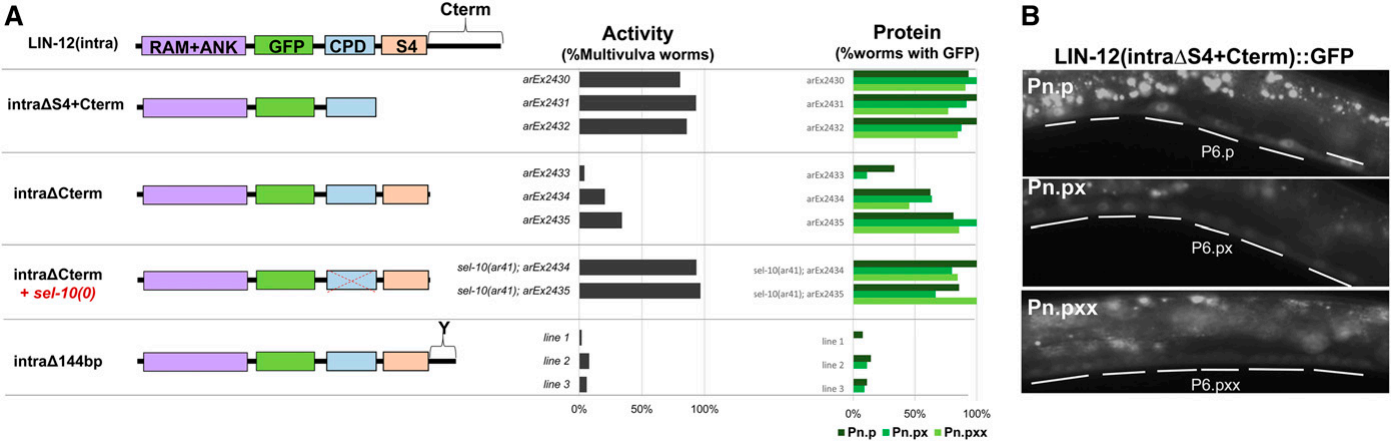
The Cterm region influences the stability of LIN-12(intra). (A) Analysis of the Cterm region. Removing the entire Cterm region in LIN-12(intraΔS4+Cterm) and LIN-12(intraΔCterm) stabilizes the intracellular domain in the VPC descendants as well as in VPCs. Deletion of the last 48 amino acids does not affect LIN-12(intra) protein stability in VPC descendants, and the presence of the first 22 amino acids of the Cterm region is sufficient for normal regulation. We term this 22 amino acid region the “Y region,” after a tyrosine moiety that is required for normal regulation (see Figure 5). (B) The LIN-12(intraΔS4+Cterm) protein is stabilized in all VPC stages. The GFP is visible in all the VPCs in Pn.p (VPCs), Pn.px (their daughters), and Pn.pxx (their granddaughters). Images were taken at 600 msec exposure time. CPD, Cdc4 phosphodegron; GFP, green fluorescent protein.

**Figure 5:**
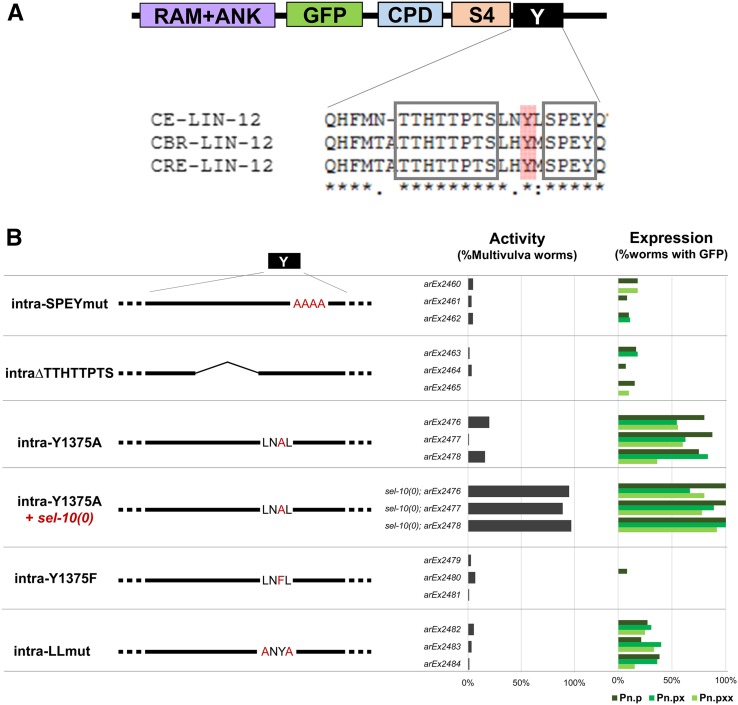
Identification of a sequence that is conserved in LIN-12 of multiple *Caenorhabditis* species and influences LIN-12(intra) stability. (A) Conserved regions within the Cterm. The required region for downregulation in the VPC descendants is located in the first 22 amino acids of the Cterm. Alignment of the *C. elegans*, *C. briggsae*, and *C. remanei* LIN-12 sequences show two well-conserved sequences in that region, “TTHTTPTS” and “SPEY,” and the conserved tyrosine highlighted in red, analyzed in (B). (B) Analysis of conserved regions shown in (A). The two well-conserved regions that contain amino acids that could serve as sites of posttranslational modification, “TTHTTPTS” and “SPEY,” in the Cterm are not required for negative regulation of LIN-12(intra) activity or stability of the protein in VPC and VPC descendants. The tyrosine between the two regions, Y1375, appears to be structurally important for downregulation rather than for a modification such as phosphorylation because, although the alanine substitution mutant LIN-12(intra-Y1375A) is stabilized in VPCs and its descendants, the more conservative phenylalanine substitution mutant LIN-12(intra-Y1375F) is not. The region around the tyrosine also influences LIN-12(intra) stability, as LIN-12(intra-LLmut) is also stabilized in VPCs and their descendants [Pn.p (VPCs), Pn.px (their daughters), and Pn.pxx (their granddaughters)], but to a lesser extent than Y1375A. CPD, Cdc4 phosphodegron; GFP, green fluorescent protein.

Since mutation of the CPD and S4 regions did not stabilize LIN-12(intra) in VPC descendants, we inferred that other sequences absent from LIN-12(intraΔP) account for its stabilization. Indeed, deletion of either the S4+Cterm or just the Cterm region resulted in significant stabilization in VPC descendants, indicating that the Cterm region negatively regulates the stability of LIN-12(intra) (Figure 4, A and B). Deletion of the last 48 amino acids from the carboxy-terminus did not cause a Multivulva phenotype or visible protein accumulation (Figure 4A), indicating that this portion of the Cterm region does not negatively regulate LIN-12(intra) stability, and that determinants affecting stability instead reside in a 22 amino acid region we refer to here as the “Y” region (Figure 4A). The Y region of LIN-12 does not display overt conservation with Notch proteins from other phyla; however, comparison of LIN-12 from three different *Caenorhabditis* species identified well-conserved motifs within the Y region to test as potential regulatory sites (Figure 5A).

We performed mutagenesis of two conserved stretches containing candidate amino acids for posttranslational modification: (i) we substituted alanines for the four well-conserved amino acids “SPEY” and (ii) we generated in-frame deletion of the well-conserved sequence “TTHTTPTS” (Figure 5A). In both cases, the mutant proteins had low constitutive activity and were degraded like wild-type LIN-12(intra) (Figure 5B).

In contrast, when we mutated the well-conserved tyrosine Y1375 located between these two sequences to alanine, we observed stabilization of LIN-12(intra-Y1375A) protein in VPCs and their descendants. To test if Y1375 serves as a potential phosphorylation site, we made the more conservative Y1375F mutation, and found that the resulting mutant LIN-12(intra-Y1375F) protein is not stabilized (Figure 5B). Based on these results, we infer that posttranslational modification of Y1375 is not important; rather, this residue may play a structural role or mediate protein interactions that govern LIN-12(intra) stability. Additional support for this inference comes from the observation that mutation of the two conserved leucines flanking Y1375 to alanine also stabilized LIN-12(intra-L1373A,L1376A) in VPCs and their descendants, albeit to a lesser extent that LIN-12(intra-Y1375A) (Figure 5B). These results suggest that: (i) this conserved region of the Cterm is important for its structure and (ii) the Cterm region influences negative regulation of LIN-12(intra) levels in VPCs and their descendants.

### Downregulation of the LIN-12 intracellular domain in VPCs and their descendants requires assembly of the nuclear complex

**Figure 6:**
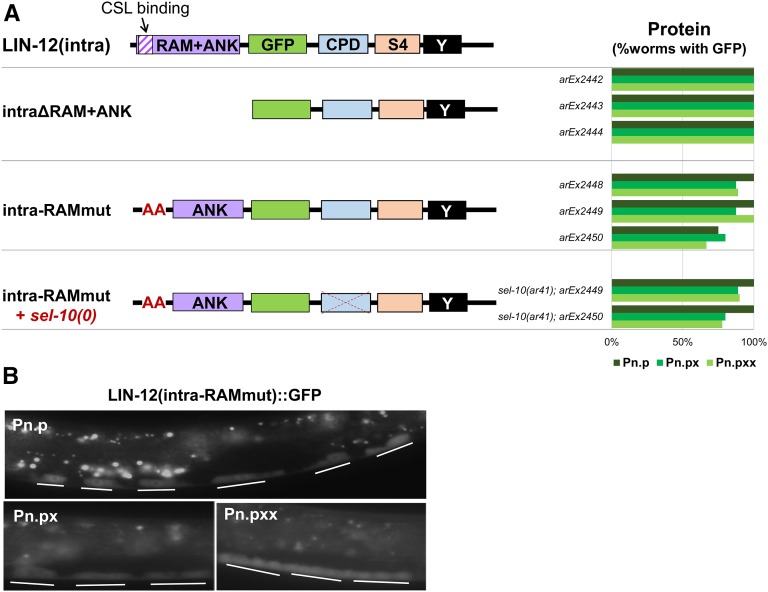
Downregulation of the LIN-12 intracellular domain requires assembly of the nuclear complex. (A) Analysis of mutations affecting the RAM domain. The RAM domain contains a tetrapeptide site required for LAG-1/CSL to bind to LIN-12(intra) and subsequently form a nuclear complex (Wilson and Kovall 2006). Deletion of the RAM+ANK or mutation of the LAG-1/CSL binding site prevent LIN-12(intra) from associating with the nuclear complex, which abrogates LIN-12(intra) activity and stabilizes LIN-12(intra) protein in the VPC and VPC descendants [Pn.p (VPCs), Pn.px (their daughters), and Pn.pxx (their granddaughters)]. (B) Photomicrographs showing stabilization resulting from mutation of the RAM domain. CPD, Cdc4 phosphodegron; GFP, green fluorescent protein.

Although deletion of the PEST sequence stabilizes LIN-12(intra) in VPCs and their descendants, a deletion of the amino terminus that leaves the PEST sequence intact is insufficient to promote turnover in these cells (Figure 6A), indicating that its ability to do so may depend on the context in which it is located or some other property of LIN-12(intra).

In mammalian cells, phosphorylation of the Notch1 intracellular domain can occur in the nuclear complex when it is associated with target DNA, recruiting Fbw7 and leading to destruction that ends that transcription event (Fryer *et al.* 2004). However, the characterized phosphorylation sites do not correspond to the canonical CPD phosphorylation sites that recruit Fbw7 in other substrates (Welcker and Clurman 2008) or to the serines of the S4 region, and are not conserved in LIN-12. Nevertheless, we tested the possibility that LIN-12(intra) must be in the nuclear complex to be downregulated by mutating the VWMP tetrapeptide motif in the RAM domain of LIN-12(intra), which anchors it to LAG-1/CSL in the nuclear complex (Figure 6) (Wilson and Kovall 2006). Mutation of the W and P moieties of this motif to alanine would be predicted to result in an inactive LIN-12 intracellular domain since it cannot bind to LAG-1 to form the nuclear complex. Indeed, this LIN-12(intra-RAMmut) mutant does not cause a Multivulva phenotype, even in a *sel-10(0)* background (Figure 6A). The LIN-12(intra-RAMmut) protein is stable in the VPCs and VPC descendants (Figure 6B), suggesting that LIN-12(intra) has to be in the nuclear complex in order to be degraded.

### Mutational uncoupling of stability and activity

As described above, wild-type LIN-12(intra) has low constitutive activity and stability in VPCs, and loss of *sel-10* concomitantly enhances its constitutive activity and stability. A similar correlation between enhanced constitutive activity (Multivulva phenotype) and stability (visible GFP) is seen for most of the mutant forms we describe in this study. However, there are two notable exceptions: (i) when the carboxy-terminal region after the S4 domain is deleted [LIN-12(intraΔCterm] (Figure 4A), and (ii) when the conserved tyrosine has been mutated [LIN-12(intra-Y1375A)] (Figure 5B). In both of these cases, there appears to be considerable stabilization of the mutant LIN-12(intra-X) protein without increasing its constitutive activity, indicating that LIN-12(intra) function is compromised by these alterations in the Cterm region. Additionally, when we removed *sel-10(0)* activity from each of these two mutants, we observed a strong Multivulva phenotype (Figure 4A and Figure 5B), suggesting that there may be roles for the carboxy-terminal region and *sel-10* independent of their role in LIN-12(intra) stability.

## Discussion

We have shown that there are distinct mechanisms for temporal regulation of the stability of the LIN-12 intracellular domain in VPCs and their descendants; SEL-10/Fbw7 and the PEST sequence, including the individual CPD and S4 domains within it, negatively regulate stability of LIN-12(intra) in VPCs, but not in VPC descendants. We have also provided evidence that sequences within the carboxy-terminal region outside of the PEST sequence influence stability of LIN-12(intra) in VPCs and their descendants. This “Y region” is named for a tyrosine moiety that is found in LIN-12 and its orthologs in other nematode species, as well as its paralog GLP-1 in *C. elegans*, and our mutational analysis suggests that it contributes to the structure or mediates protein interactions rather than providing a site for posttranslational modification *per se*. Furthermore, we have provided evidence that downregulation of LIN-12(intra) in both VPCs and their descendants requires its association with the nuclear complex.

Most of our manipulations resulted in a consistent correlation between LIN-12(intra) activity and stability in VPCs: low constitutive activity and low stability are coupled, and high constitutive activity and high stability are coupled. However, certain mutations in the Cterm region uncoupled these properties, resulting in increased stability without increased activity. Since an intact nuclear complex also appears to be a prerequisite for downregulation, it is tempting to speculate that the structure of the abnormal Cterm region reduces the assembly of the nuclear complex. Furthermore, although the Cterm region is not conserved in terms of primary amino acid sequence between nematode and mammalian Notch proteins, our evidence favoring a structural role rather than a specific sequence modification raises the possibility that there will be a similar influence in the sequence of the carboxy-terminus in other Notch proteins, as there are precedents for structural similarity between *C. elegans* and mammalian proteins even when primary sequence divergence is too great to detect it (Liu *et al.* 2008).

When the LIN-12(intraΔCterm) and LIN-12(intra-Y1375A) mutants, which are stable but have low constitutive activity, are placed into a *sel-10* null mutant background, their constitutive activity is increased so as to cause a Multivulva phenotype. This behavior may simply reflect stabilization of the abnormal LIN-12(intra) structure to promote its ability to associate with the nuclear complex. However, we speculate that SEL-10 may have a role other than promoting the degradation of LIN-12(intra) *per se*. An intriguing precedent for an alternative role is provided by the observation that Fbw7 has more than one role in the degradation of another of its substrates, Cyclin E. First, Fbw7 serves as a cofactor of the prolyl *cis*-trans isomerase Pin1 for isomerizing a bond in the Cyclin E CPD, with such modification being prerequisite for Fbw7 binding to promote ubiquitination of Cyclin E (Yeh *et al.* 2006). Another potential precedent is provided by the finding that another Fbw superfamily member, β-TrCp1/Fbw1a, is part of a transcription complex that includes its substrate β-catenin to enhance transcription of target genes rather than degradation (Semplici *et al.* 2002).

## Supplementary Material

Supplemental Material
