# Supplementary material for: Determinants in the LIN-12/Notch Intracellular Domain That Govern Its Activity and Stability During Caenorhabditis elegans Vulval Development
Source: G3 (Bethesda). 2016 Sep 16;6(11):3663–70. doi: 10.1534/g3.116.034363 (PMC5100865; doi:10.1534/g3.116.034363)
Supplement: Supplemental Material [file supp_6_11_3663__index.html]

Determinants in the LIN-12/Notch Intracellular Domain That Govern Its Activity and Stability During Caenorhabditis elegans Vulval Development — Supplemental Material 

# Determinants in the LIN-12/Notch Intracellular Domain That Govern Its Activity and Stability During *Caenorhabditis elegans* Vulval Development

## Supplemental Material Deng and Greenwald, 2016

**Files in this Data Supplement:**

- Table S1 - Plasmids generated in this study. (.pdf, 37 KB)
- Table S2 - Plasmids and primers used to make constructs in Table 1. (.pdf, 30 KB)
- Table S3 - Strains and transgenes generated in this study. (.pdf, 50 KB)
