# Supplementary material for: Determinants in the LIN-12/Notch Intracellular Domain That Govern Its Activity and Stability During Caenorhabditis elegans Vulval Development
Source: G3 (Bethesda). 2016 Sep 16;6(11):3663–70. doi: 10.1534/g3.116.034363 (PMC5100865; doi:10.1534/g3.116.034363)
Supplement: Supplemental Material [file supp_g3.116.034363_TableS1.pdf]

Table S1. Plasmids generated in this study.

Plasmid #	Construct
p935	lin-31p::lin-12(intra)::GFP
p937	lin-31p::lin-12(intra-CPDmut)::GFP
p938	lin-31p::lin-12(intraΔ(S4+Cterm))::GFP
p939	lin-31p::lin-12(intraΔCterm)::GFP
p941	lin-31p::lin-12(intraΔPEST)::GFP
p942	lin-31p::GFP::lin-12(intraΔ(RAM+ANK))::GFP
p944	lin-31p::lin-12(intra-RAMmut)::GFP
p945	lin-31p::lin-12(intra[3])::GFP
p946	lin-31p::lin-12(intra-S4mut)::GFP
p947	lin-31p::lin-12(intra[2])::GFP
p948	lin-31p::lin-12(intraΔ144bp)::GFP
p949	lin-31p::lin-12(intra-SPEYMut)::GFP
p951	lin-31p::lin-12(intraΔTTHTTPTS)::GFP
p960	lin-31p::lin-12(intra-Y1375A)::GFP
p963	lin-31p::lin-12(intra-Y1375F)::GFP
p964	lin-31p::lin-12(intra-LLmut)::GFP

For transgenes generated with these constructs, see Table S3.
