# Supplementary material for: Determinants in the LIN-12/Notch Intracellular Domain That Govern Its Activity and Stability During Caenorhabditis elegans Vulval Development
Source: G3 (Bethesda). 2016 Sep 16;6(11):3663–70. doi: 10.1534/g3.116.034363 (PMC5100865; doi:10.1534/g3.116.034363)
Supplement: Supplemental Material [file supp_g3.116.034363_TableS2.pdf]

Table S2. Plasmids and primers used to make constructs in Table 1.

oYD	Plasmid Used	Sequence
oYD45	All	GCTCTTCATCAAGCGGCTGC
oYD47	p941	GCTGGCGGCCGCCGACCTTTGTATAGTTCATCCATGCC
oYD48	All	AAAGATCTTTGTCTCGAGAATCCATGC
oYD52	p941	GCTGGCGGCCGCTCAACCTTTGTATAGTTCATCCATGCC
oYD53	p938	GCTGGCGGCCGCTCAATCCGATGGTGGAGGTGTCAG
oYD54	p939	GCTGGCGGCCGCTCACTGTGGTGACGGTGTTGACG
oYD56	p942	CGCCAGATCTACCGGTAGAAAAAATGAGTAAAGGAGAAGAACTTTTCACTGG
oYD57	p946	gatggaGcaGcgGcaGcaGcgGcaccacagcattttatgaataccactcatactacacc
oYD58	p946	ctgtggtgCcgCtgCtgCcgCtgCtccatccgatggtggaggtgt
oYD59	p947	ccgaaccagctcattattttATGAGTAAAGGAGAAGAACTTTTCACTGGAG
oYD60	p947	AGTTCTTCTCCTTTACTCATaaaataatgagctggttcggagtatcgc
oYD61	p947	GCTGGCGGCCGCtcaACCTTTGTATAGTTCATCCATGCCATGTG
oYD63	p945	cttcgacttgtctgtttcttcggagcACCTTTGTATAGTTCATCCATGCCATGTG
oYD64	p945	agtctcgagaattggaagctgactctttttttgcttcgacttgtctgtttcttcggagcACCTTTGTATAGTTCATCCATGCCATGTG
oYD65	p944	acgcctctgtcGCgGCgGcacctatggagaatgaggagaagaatcgg
oYD66	p944	gccagatctatgggaaatcggacaaggaaacgtcgaatgatcaacgcctctgtcGCgGCgGcacctatggagaatgaggagaagaatcgg
oYD69	p949	ttgaGCtGctgCgGCcaaatagttcaaactcgtcggtgtagtatg
oYD70	p949	ttgGCcGcagCaGCtcaaactgaagctggaagctcagaag
oYD71	p948	gctggcggccgctcattgatattctgggctcaaatagttcaaactcg
oYD82	p951	cattttatgaatttgaactatttgagcccagaatatcaaactg
oYD83	p951	caaatagttcaaattcataaaatgctgtggtgacggtg
oYD96	p960	gacgagtttgaacGCtttgagcccagaatatcaaactgaagc
oYD97	p960	ttctgggctcaaaGCgttcaaactcgtcggtgtagtatgagtg
oYD106	p963	gacgagtttgaactTtttgagcccagaatatcaaactgaagc
oYD107	p963	ttctgggctcaaaAagttcaaactcgtcggtgtagtatgagtg
