# Supplementary material for: Determinants in the LIN-12/Notch Intracellular Domain That Govern Its Activity and Stability During Caenorhabditis elegans Vulval Development
Source: G3 (Bethesda). 2016 Sep 16;6(11):3663–70. doi: 10.1534/g3.116.034363 (PMC5100865; doi:10.1534/g3.116.034363)
Supplement: Supplemental Material [file supp_g3.116.034363_TableS3.pdf]

Table S3. Strains and transgenes generated in this study.

Name	Genotype	Construct	Figure
GS7374	*pha-1(e2123); arEx1992*	canonical intra::GFP	2
GS7375	*pha-1(e2123); arEx1993*	canonical intra::GFP	2
GS8310	*pha-1(e2123); arEx2427*	intra-CPDmut	3
GS8311	*pha-1(e2123); arEx2428*	intra-CPDmut	3
GS8312	*pha-1(e2123); arEx2429*	intra-CPDmut	3
GS8313	*pha-1(e2123); arEx2430*	intraΔS4+Cterm	4
GS8314	*pha-1(e2123); arEx2431*	intraΔS4+Cterm	4
GS8315	*pha-1(e2123); arEx2432*	intraDS4+Cterm	4
GS8316	*pha-1(e2123); arEx2433*	intraΔCterm	4
GS8317	*pha-1(e2123); arEx2434*	intraΔCterm	4
GS8318	*pha-1(e2123); arEx2435*	intraΔCterm	4
GS8323	*pha-1(e2123); arEx2439*	intraΔP	2
GS8324	*pha-1(e2123); arEx2440*	intraΔP	2
GS8325	*pha-1(e2123); arEx2441*	intraΔP	2
GS8326	*pha-1(e2123); arEx2442*	intraΔRAM+ANK	6
GS8327	*pha-1(e2123); arEx2443*	intraΔRAM+ANK	6
GS8328	*pha-1(e2123); arEx2444*	intraΔRAM+ANK	6
GS8332	*pha-1(e2123); arEx2448*	intra-RAMmut	6
GS8333	*pha-1(e2123); arEx2449*	intra-RAMmut	6
GS8334	*pha-1(e2123); arEx2450*	intra-RAMmut	6
GS8335	*pha-1(e2123); arEx2451*	intra[3]	2
GS8336	*pha-1(e2123); arEx2452*	intra[3]	2
GS8337	*pha-1(e2123); arEx2453*	intra[3]	2
GS8338	*pha-1(e2123); arEx2454*	intra-S4mut	3
GS8339	*pha-1(e2123); arEx2455*	intra-S4mut	3
GS8340	*pha-1(e2123); arEx2456*	intra-S4mut	3
GS8341	*pha-1(e2123); arEx2457*	intra[2]	2
GS8342	*pha-1(e2123); arEx2458*	intra[2]	2
GS8343	*pha-1(e2123); arEx2459*	intra[2]	2
GS8344	*pha-1(e2123); arEx2460*	intra-SPEYmut	5
GS8345	*pha-1(e2123); arEx2461*	intra-SPEYmut	5
GS8346	*pha-1(e2123); arEx2462*	intra-SPEYmut	5

GS8347	*pha-1(e2123); arEx2463*	intraΔTTHTTPTS	5
GS8348	*pha-1(e2123); arEx2464*	intraΔTTHTTPTS	5
GS8349	*pha-1(e2123); arEx2465*	intraΔTTHTTPTS	5
GS8360	*pha-1(e2123); arEx2476*	intra-Y1375A	5
GS8361	*pha-1(e2123); arEx2477*	intra-Y1375A	5
GS8362	*pha-1(e2123); arEx2478*	intra-Y1375A	5
GS8363	*pha-1(e2123); arEx2479*	intra-Y1375F	5
GS8364	*pha-1(e2123); arEx2480*	intra-Y1375F	5
GS8365	*pha-1(e2123); arEx2481*	intra-Y1375F	5
GS8366	*pha-1(e2123); arEx2482*	intra-LLmut	5
GS8367	*pha-1(e2123); arEx2483*	intra-LLmut	5
GS8368	*pha-1(e2123); arEx2484*	intra-LLmut	5

All constructs express forms of LIN-12(intra) tagged with GFP. Except for “intra[2]” and “intra[3],” the GFP tag is inserted as in the canonical “intra::GFP” as described in the text and Fig. 2.
